# Voluntary running exercise modifies astrocytic population and features in the peri-infarct cortex

**DOI:** 10.1016/j.ibneur.2023.02.004

**Published:** 2023-02-22

**Authors:** Natsumi Yamaguchi, Toshinori Sawano, Jin Nakatani, Akiko Nakano-Doi, Takayuki Nakagomi, Tomohiro Matsuyama, Hidekazu Tanaka

**Affiliations:** a Pharmacology Laboratory, Department of Biomedical Sciences, College of Life Sciences, Ritsumeikan University, 1–1-1 Noji-Higashi, Kusatsu, Shiga 525–8577, Japan; b Ritsumeikan Advanced Research Academy, 1 Nishinokyo-Suzaku-cho, Nakagyo-ku, Kyoto 604–8520, Japan; c Institute for Advanced Medical Sciences, Hyogo College of Medicine, 1–1 Mukogawacho, Nishinomiya 663–8501, Japan; d Department of Therapeutic Progress in Brain Diseases, Hyogo College of Medicine, 1–1 Mukogawacho, Nishinomiya 663–8501, Japan

**Keywords:** ACSA-2, astrocyte cell surface antigen-2, BrdU, 5-bromo-2′-deoxyuridine, DEG, differentially expressed gene, EDTA, ethylenediaminetetraacetic acid, FBS, fetal bovine serum, GO, gene ontology, GFAP, glial fibrillary acidic protein, GST-π, glutathione S-transferase-π, Gstp1, glutathione S-transferase, pi 1, Gstp2, glutathione S-transferase, pi 2, Iba1, ionized calcium-binding adapter molecule 1, Ig, immunoglobulin, Lcn2, lipocalin 2, MCAO, middle cerebral artery occlusion, PBS, phosphate-buffered saline, PFA, 4% paraformaldehyde, POD, post-operative day, qPCR, quantitative polymerase chain reaction, TUNEL, terminal deoxynucleotidyl transferase-mediated dUTP nick 3’-end labeling, Vegfa, vascular endothelial growth factor A, Vtn, vitronectin, Cerebral ischemia, Voluntary running exercise, Astrocytes, Transcriptome, Proliferation

## Abstract

Rehabilitative exercise following a brain stroke has beneficial effects on the morphological plasticity of neurons. Particularly, voluntary running exercise after focal cerebral ischemia promotes functional recovery and ameliorates ischemia-induced dendritic spine loss in the peri-infarct motor cortex layer 5. Moreover, neuronal morphology is affected by changes in the perineuronal environment. Glial cells, whose phenotypes may be altered by exercise, are known to play a pivotal role in the formation of this perineuronal environment. Herein, we investigated the effects of voluntary running exercise on glial cells after middle cerebral artery occlusion. Voluntary running exercise increased the population of glial fibrillary acidic protein-positive astrocytes born between post-operative days (POD) 0 and 3 on POD15 in the peri-infarct cortex. After exercise, transcriptomic analysis of post-ischemic astrocytes revealed 10 upregulated and 70 downregulated genes. Furthermore, gene ontology analysis showed that the 70 downregulated genes were significantly associated with neuronal morphology. In addition, exercise reduced the number of astrocytes expressing lipocalin 2, a regulator of dendritic spine density, on POD15. Our results suggest that exercise modifies the composition of astrocytic population and their phenotype.

## Graphical Abstract

**Figure:**
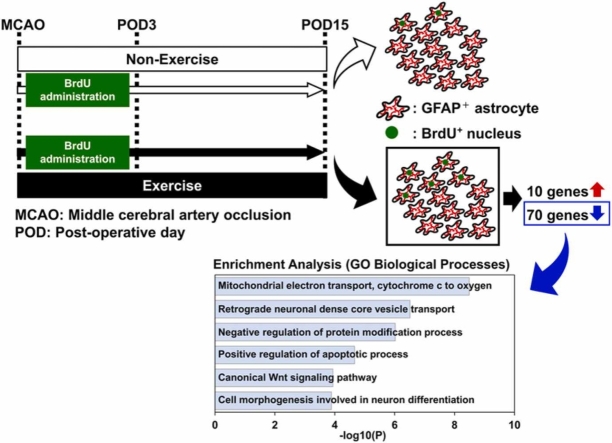

## Introduction

Ischemic stroke occurs because of an inadequate blood supply to the brain, resulting in the induction of tissue necrosis in the ischemic core. Moreover, the size and location of the necrotic area influences the extent of the resulting neurological impairment. Although neuronal loss is irreversible given the limited neurogenic capacity of the central nervous system, some forms of neuronal plasticity outside the ischemic core allow for functional recovery (Biernaskie and Corbett, 2001, Jones and Schallert, 1992). We previously revealed that voluntary running exercise after focal cerebral ischemia promotes functional recovery and ameliorates ischemia-induced dendritic spine loss in the peri-infarct motor cortex layer 5 (Yamaguchi et al., 2021).

However, since neurons are damaged in the peri-infarct core (Enright et al., 2007), a supporting environment is needed to enhance beneficial neuronal plasticity. Additionally, synaptic remodeling is influenced by the perineuronal environment (Eroglu and Barres, 2010, Song and Dityatev, 2018), in which glial cells (astrocytes, oligodendrocytes, and microglia) are important components. Cerebral ischemia enhances glial proliferation (Li et al., 2014, Sawano et al., 2019, Sawano et al., 2015, Wada et al., 2016), which can induce changes in the perineuronal environment. Furthermore, exercise can affect the number, morphology, and function of glial cells (Andoh et al., 2019, Lundquist et al., 2019, Zheng et al., 2019). Thus, glial cells may be targeted by rehabilitative exercise following cerebral ischemia.

In this study, we demonstrated that exercise increases the population of GFAP-positive astrocytes born between post-operative days (POD) 0 and 3 on POD15 accompanied by functional changes in astrocytes, including reduction in lipocalin 2 (Lcn2) expression.

## Experimental procedures

### Animals

Male C.B-17/Icr-^+/+^Jcl mice were purchased from CLEA Japan (Tokyo, Japan). Eighty-one mice were divided into three groups: sham (3 mice), middle cerebral artery occlusion (MCAO) + non-exercise (39 mice), and MCAO + exercise (39 mice). Four MCAO + non-exercise mice and five MCAO + exercise mice died owing to a reduction in food intake that was caused by surgical invasion of the masticatory muscles; hence, these mice were excluded from the experiments. One MCAO + exercise mouse was excluded from the experiments because of its unusual vascular structure. The detailed number of mice in each experiment is indicated in each figure legend. All animal experiments were approved by the Animal Care and Use Committee of Ritsumeikan University, Biwako Kusatsu Campus. We used as few animals as possible and always minimized their suffering.

### Middle cerebral artery occlusion

C.B-17/Icr-^+/+^Jcl mice display low variability in cerebral vascular structures (Taguchi et al., 2010). Thus, we performed MCAO in 7-week-old C.B-17/Icr-^+/+^Jcl mice to induce reproducible cerebral infarction as previously described (Taguchi et al., 2010, Yamaguchi et al., 2021). Briefly, mice were anesthetized with a mixture of medetomidine (0.3 mg/kg; Nippon Zenyaku Kogyo, Fukushima, Japan), midazolam (4.0 mg/kg; Sandoz, Tokyo, Japan), and butorphanol tartrate (5.0 mg/kg; Meiji Seika Pharma, Tokyo, Japan) and were placed in semi-supination. The skin between the left eye and left ear was incised, and the cranial bone was drilled to approach the MCA. The exposed MCA was electrically occluded by using a bipolar coagulator (MS-50; MERA, Tokyo, Japan). After surgery, the mice were allowed to recover from anesthesia on a heating mat. The sham group was treated similarly but without MCAO. Crushed chow and water were provided ad libitum.

### Voluntary running exercise

For acclimatization to running exercise, mice of all three groups were housed in cages with a running wheel for 1 week as pre-training prior to MCAO. The MCAO + exercise group mice were exposed to a running wheel after MCAO for 15 days, 24 h/day, as previously described (Yamaguchi et al., 2021). One rotation was converted to a running distance of 35.168 cm. We recorded the running distance every 24 h after MCAO. After the MCAO procedure, the mice in the sham and MCAO + non-exercise groups were housed in cages without running wheels.

### 5-bromo-2′-deoxyuridine administration

To detect newborn cells after ischemia, 5-bromo-2′-deoxyuridine (BrdU; FUJIFILM Wako, Osaka, Japan) was administered to each group via intraperitoneal injection (50 mg/kg/day) immediately after MCAO and on POD1, 2, and 3.

### Immunohistochemistry

Mice were transcardially perfused with 4% paraformaldehyde (PFA) under deep isoflurane anesthesia. The brains were removed and post-fixed two overnights in PFA, followed by transfer into a 30% sucrose solution for cryoprotection. The brains were cut into consecutive 30 µm-thick coronal sections using a Leica CM1860 cryostat (Leica Microsystems GmbH, Wetzlar, Germany). The sections were blocked in 5% goat serum/0.5% Triton X-100/0.05% sodium azide in phosphate-buffered saline (PBS) for 1 h and then incubated with following primary antibodies in antibody dilution buffer (1% goat serum/0.1% Triton X-100/0.05% sodium azide in PBS) at 4 ºC for 16 h: rat anti-BrdU (BU1/75 [ICR1], 1:1000; Abcam, Cambridge, UK, Cat# ab6326, RRID: AB_305426) for BrdU-incorporated cells, rat anti-Ki67 (SolA15, 1:500; Thermo Fisher Scientific, Cat# 14–5698–82, RRID:AB_10854564) for proliferative cells, rabbit anti-ionized calcium-binding adapter molecule 1 (anti-Iba1, 1:500; FUJIFILM Wako, Osaka, Japan, Cat# 019–19741, RRID: AB_839504) for microglia, rabbit anti-glutathione S-transferase-π (anti-GST-π, 1:1000; MBL, Tokyo, Japan, Cat# Code No. 312, RRID: AB_591792) for oligodendrocytes, mouse anti-glial fibrillary acidic protein (anti-GFAP, 2E1. E9, 1:500; BioLegend, San Diego, CA, USA, Cat# 644702, RRID: AB_2294566), rabbit anti-GFAP (1:2000; Agilent, Santa Clara, CA, USA, Cat# Z0334, RRID: AB_10013382) for astrocytes, and rat anti-Lcn2 (1:100; BioLegend San Diego, CA, USA, Cat# 661502, RRID: AB_2563987). After three washes in PBS, the sections were reacted with the following appropriate secondary antibodies in antibody dilution buffer at 20 ºC for 3 h: biotin-labeled anti-rat immunoglobulin G (anti-rat IgG, 1:2000; KPL, Milford, MA, USA, Cat# 112–065–003, RRID: AB_2338168), Alexa Fluor 488-labeled anti-rat IgG (1:2000; Abcam, Cat# ab150165, RRID:AB_2650997), Alexa Fluor Plus 488-labeled anti-rabbit IgG (1:2000; Thermo Fisher Scientific, Waltham, MA, USA, Cat# A32790, RRID:AB_2762833), Alexa Fluor 546-labeled anti-rabbit IgG (1:2000; Thermo Fisher Scientific, Cat# A10040, RRID: AB_2534016), Alexa Fluor Plus 647-labeled anti-rabbit IgG (1:2000; Thermo Fisher Scientific, Cat# A32795, RRID: AB_2762835), and Alexa Fluor 647-labeled anti-mouse IgG (1:2000; Thermo Fisher Scientific, Cat# A21236, RRID: AB_2535805). Biotin-labeled secondary antibodies were visualized using streptavidin 488 (1:2000; Thermo Fisher Scientific, Cat# S11223). To detect BrdU, the sections were treated with 1N HCl for 30 min at 37 ºC and for 30 min at 25 ºC before immunohistochemical staining. Nuclei were visualized using DAPI (1:5000; Sigma, Darmstadt, Germany, Cat# D9542).

For Nissl staining, the sections were incubated with 0.025% (w/v) thionin acetate in 0.1N sodium acetate buffer at 37 ºC for 30 min, subsequently dehydrated consecutively in 70% and 100% ethanol, and cleared in Hemo-De (Falma, Tokyo, Japan). Finally, the sections were mounted on glass slides and embedded in Marinol (Muto pure chemicals, Tokyo, Japan).

### Terminal deoxynucleotidyl transferase-mediated dUTP nick 3’-end labeling (TUNEL) assay

To detect dying cells, GFAP- and DAPI-stained sections were subjected to TUNEL assay using the DeadEnd Fluorometric TUNEL System (Promega, Madison, WI, USA). Briefly, the sections were incubated in equilibration buffer for 15 min, subsequently incubated with fluorescein-12-dUTP and terminal deoxynucleotidyl transferase for 60 min at 37 ºC.

### Image acquisition

Coronal sections of the bregma—between 1.98 and 1.70 mm—were analyzed. The sections were scanned and reconstructed using a confocal microscope (FLUOVIEW FV10i; OLYMPUS, Tokyo, Japan) with a 60 × objective lens (OLYMPUS Plan Apo, NA = 1.4) or an ECLIPSE Ti2 microscope (Nikon, Tokyo, Japan) attached to a confocal unit (MAICO; HAMAMATSU PHOTONICS, Shizuoka, Japan) with a 40 × objective lens (Nikon Plan Apo, NA = 1.0). The infarct area was identified as the area where the nuclei shrank. To analyze a 424 µm × 424 µm area next to the infarct per section, images were captured in a field of view of 212 µm × 212 µm at four positions, and one section per brain was analyzed. To analyze a 450 µm × 400 µm area adjacent to the infarct per section, images were captured in a field of view of 150 µm × 200 µm at six positions, and three sections per brain were analyzed.

### Cell counting

A blinded experimenter counted the nuclei (DAPI, BrdU, and TUNEL) to determine the number of each cell type. The data are presented as the number of cells per field (424 µm × 424 µm or 450 µm × 400 µm).

### Measurement of Iba1-stained area

Image processing and analysis were performed using Fiji software (http://fiji.sc/ Fiji). Iba1-stained images were converted into 8-bit images, which were binarized using the Default algorithm; Iba1-stained areas are shown as black and automatically measured.

### Magnetic sorting of astrocytes

On POD15, astrocytes were sorted from the ipsilateral cortices. Two or three mice were used in each experiment. Tissue in 20 U/mL papain and 100 µg/mL DNase in Hanks’ balanced salt solution with 10 mM 2-[4-(2-Hydroxyethyl)-1-piperazinyl] ethanesulfonic acid-NaOH (pH 7.4) was dissociated twice using an 18G needle. This suspension was incubated at 37 ºC for 30 min and dissociated twice using 23G and 27G needles. The cell suspension was further isolated using a 70 µm cell strainer. The strainer was washed with 10 mL of PBS containing 2% fetal bovine serum (FBS) and 1 mM ethylenediaminetetraacetic acid (EDTA). The cells in PBS solution were centrifuged at 300 × *g* for 3 min at 4 ºC. The cells were resuspended in 500 µL of PBS containing 2% FBS and 1 mM EDTA. The cell suspension was centrifuged at 300 × *g* for 3 min at 4 ºC and the supernatant was discarded. The cells were suspended in 34 µL of PBS containing 2% FBS, 1 mM EDTA, and 66 µL of 30 µg/mL biotinylated anti-astrocyte cell surface antigen-2 antibody (anti-ACSA-2; Miltenyi Biotec B.V. & Co. KG, Bergisch Gladbach, Germany, Cat# 130–101–879, RRID: AB_2651191). After 15 min, 4 mL of PBS containing 2% FBS and 1 mM EDTA was added, and the solution was centrifuged at 300 × *g* for 5 min at room temperature after pipetting. The supernatant was discarded, and the cells were suspended in 100 µL of PBS containing 2% FBS and 1 mM EDTA. Next, 10 µL of Mojosort Streptavidin Nanobeads (BioLegend, San Diego, CA, USA) were added and allowed to react for 15 min followed by the addition of 2.5 mL of PBS containing 2% FBS and 1 mM EDTA by pipetting. The tubes were placed on an EasyEights EasySep Magnet (VERITAS, Santa Clara, CA, USA) for 10 min, after which the supernatant was discarded. This procedure was repeated three times, and the magnetically sorted cells were suspended in 200 µL of PBS containing 2% FBS and 1 mM EDTA.

### Microarray

Total RNA was extracted from sorted astrocytes using the TRIZOL reagent (Thermo Fisher Scientific). Approximately 70 ng of total RNA from each of the four samples per group were mixed into one pooled sample. Approximately 200 ng of total RNA per group was analyzed using the Clariom™ S Assay (Thermo Fisher Scientific) with a contracted analytical service (Filgen, Aichi, Japan). The GCCN-SST-RMA algorithm was used for data normalization. The data are available in the NCBI Gene Expression Omnibus database (accession number GSE210674) (https://www.ncbi.nlm.nih.gov/geo/query/acc.cgi?acc=GSE210674). We eliminated genes with lower expression levels than those of the negative control. Differentially expressed genes (DEGs) were defined as genes with a log2 fold change > 1 (upregulated genes) or < −1 (downregulated genes). The downregulated genes in the MCAO + exercise group were clustered by applying gene ontology (GO) biological processes using Metascape (https://metascape.org/gp/index.html#/main/step1, accessed on June 2022). Gene identifiers were first converted into their corresponding *Mus musculus* Entrez gene IDs using the latest version of the database (last updated on 4/22/2022). Terms with a *p*-value < 0.01, minimum count of 3, and enrichment factor > 1.5 were collected and grouped into clusters.

### Quantitative polymerase chain reaction (qPCR) of astrocytic genes

Total RNA was extracted from sorted astrocytes using the TRIZOL reagent (Thermo Fisher Scientific). cDNA was prepared using ReverTra Ace qPCR RT Master Mix with gDNA Remover (TOYOBO, Osaka, Japan) from 100 ng total RNA per sample. qPCR was performed using THUNDERBIRD SYBR qPCR Mix (TOYOBO) and KOD SYBR qPCR Mix with the following gene-specific primers: *36b4* forward primer, 5′-TCATCCAGCAGGTGTTTGACA-3′; *36b4* reverse primer, 5′-GGCACCGAGGCAACAGTT-3′; *vascular endothelial growth factor A* (*Vegfa)* forward primer, 5′-GCTACTGCCGTCCGATTGAG-3′; *Vegfa* reverse primer, 5′-TGATCCGCATGATCTGCATGG-3′; *Lcn2* forward primer, 5′-CCACCACGGACTACAACCAG-3′; and *Lcn2* reverse primer, 5′-AGCTCCTTGGTTCTTCCATACA-3′. Target gene expression was normalized to that of *36b4*. All data were divided by average expression in the MCAO + exercise group.

### Statistical analyses

Data are presented as mean ± standard error of the mean. For large samples (n ≥ 5), statistical analyses were performed using Welch’s t-test in Excel version 2206 (Microsoft, Redmond, WA, USA). Statistical significance was set at *p* < 0.05. For BrdU staining, one mouse from the MCAO + non-exercise group and three mice from the MCAO + exercise group were excluded owing to poor staining quality.

## Results

### Exercise increased newborn astrocyte population after cerebral ischemia

**Fig. 1:**
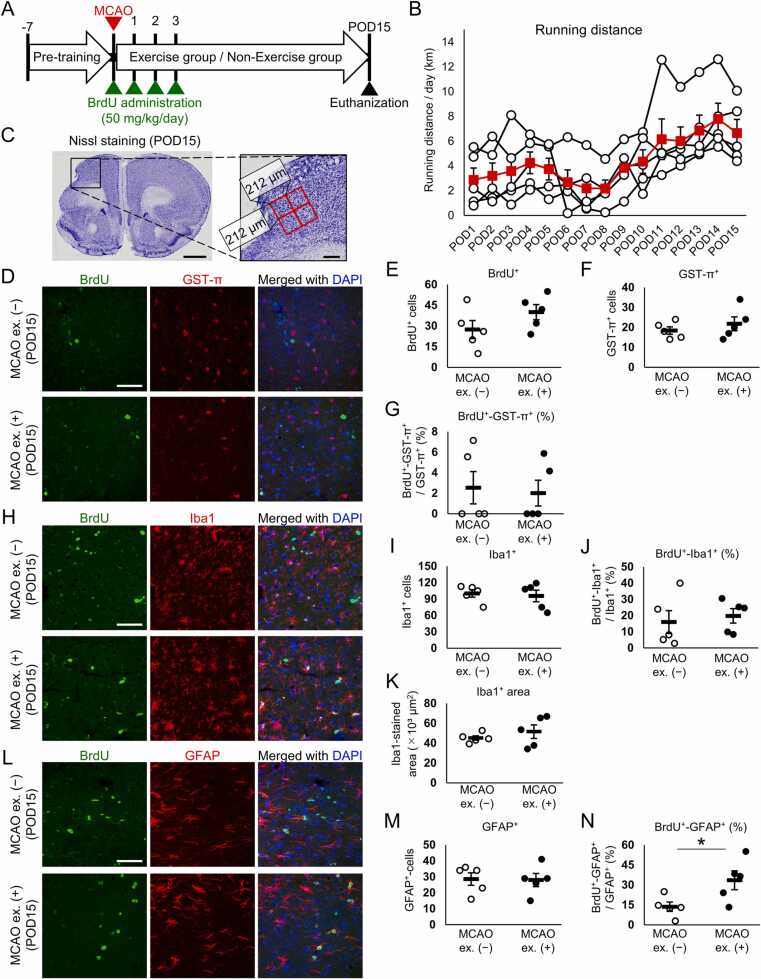
Exercise increased the population of newborn GFAP-positive cells after cerebral ischemia. (A) After pre-training (1 week), mice were divided into MCAO + non-exercise (MCAO ex. [−]) and MCAO + exercise (MCAO ex. [+]) groups and received a BrdU injection. (B) Average (red) and individual (white) running distance per 24 h after MCAO. (C) Representative Nissl staining in a POD15 mouse brain. We analyzed a 424 µm × 424 µm area next to the infarct per section by capturing four positions in a field of view of 212 µm × 212 µm. Scale bar: 1 mm (low-magnification image) and 200 µm (high-magnification image). (D) Sections were stained for BrdU and GST-π on POD15. The infarct boundary is located at the left side of the image. Scale bar: 50 µm. (E-G) The number of BrdU-positive cells (E), GST-π-positive cells (F), and the ratio of BrdU and GST-π double-positive cells to total GST-π-positive cells (G) in the MCAO + non-exercise and MCAO + exercise groups. (H) Sections were stained for BrdU and Iba1 on POD15. The infarct boundary is located at the left side of the image. Scale bar: 50 µm. (I and J) Number of Iba1-positive cells (I) and the ratio of BrdU and Iba1 double-positive cells to total Iba1-positive cells (J) in the MCAO + non-exercise and MCAO + exercise groups. (K) Iba1-stained area measure by using binarized Iba1-stained images. (L) Sections were stained for BrdU and GFAP on POD15. The infarct boundary is located at the left side of the image. Scale bar: 50 µm. (M and N) Number of GFAP-positive cells (M) and the ratio of BrdU and GFAP double-positive cells to total GFAP-positive cells (N) in the MCAO + non-exercise and MCAO + exercise groups. n = 5 mice in both the MCAO ex. (−) and MCAO ex. (+) groups. Dots and bars represent the data of individual mice and mean ± standard error of the mean, respectively. * *p* < 0.05.

Synaptic remodeling is influenced by the perineuronal environment (Eroglu and Barres, 2010, Song and Dityatev, 2018), in which glial cells are important components. On POD15, the number of dendritic spines in the peri-infarct motor cortex of the MCAO + exercise mice was larger than that of the MCAO + non-exercise mice (Yamaguchi et al., 2021). Thus, we expected that exercise would affect glial cells in the peri-infarct motor cortex on POD15. Post-ischemic glial proliferation in the peri-infarct area has been reported to peak around POD3 (Li et al., 2014, Sawano et al., 2015). Additionally, there may be differences between newborn glial cells after ischemia and residential glial cells before ischemia. Thus, we investigated the effect of exercise on the glial cell population born between POD0 and 3 on POD15. Hence, BrdU was administered on POD0 (immediately after MCAO), 1, 2, and 3. To acclimatize to running wheel exercise and initiate experiments with mice of all groups in the same pre-conditioned state, all mice were housed in cages with a running wheel for 1 week as pre-training prior to MCAO (Fig. 1A). Coronal sections of the bregma (between 1.98 and 1.70 mm) were observed to analyze the peri-infarct motor cortex.

Four of five mice ran > 1 km within 24 h after MCAO, and the running distance was gradually increased after POD8 (Fig. 1B). We analyzed the number of newborn cells between POD0 and 3 (BrdU-positive cells) and total glial cells in a 424 µm × 424 µm area next to the infarct on POD15 (Fig. 1C). The number of BrdU-positive cells in the MCAO + exercise group did not differ significantly from that in the MCAO + non-exercise group (Fig. 1D, E, H, and L). Oligodendrocytes were visualized using an anti-GST-π antibody. Exercise did not alter the number of GST-π-positive cells (Fig. 1D and F). The ratio of the number of GST-π-positive cells born between POD0 and 3 to the total number of GST-π-positive cells was low, with no significant difference between the MCAO + non-exercise and MCAO + exercise groups (Fig. 1D and G).

Microglia visualized using an anti-Iba1 antibody showed enlarged morphology (Fig. 1H), suggesting that microglial activation lasted for at least 15 days. Exercise did not affect the number of Iba1-positive cells or the ratio of Iba1-positive cells born between POD0 and 3 to the total number of Iba1-positive cells (Fig. 1H–J). Microglial activation increases Iba1-staining (Kopp et al., 2013, Sloka et al., 2013, Wang et al., 2014); however, we observed no differences in the Iba1-stained areas between the MCAO + non-exercise and MCAO + exercise groups (Fig. 1K).

Although GFAP is upregulated in reactive astrocytes (Eng and Ghirnikar, 1994), the number of GFAP-positive cells in the MCAO + exercise group did not differ from that in the MCAO + non-exercise group (Fig. 1L and M). In contrast, the ratio of GFAP-positive cells born between POD0 and 3 to the total number of GFAP-positive cells was significantly increased by exercise (*p* = 0.046) (Fig. 1N). These results indicate that voluntary running exercise increased the population of GFAP-positive astrocytes born between POD0 and 3 on POD15 without increasing the total number of GFAP-positive astrocytes.

### Exercise did not enhance astrocyte proliferation in acute phase after cerebral ischemia

**Fig. 2:**
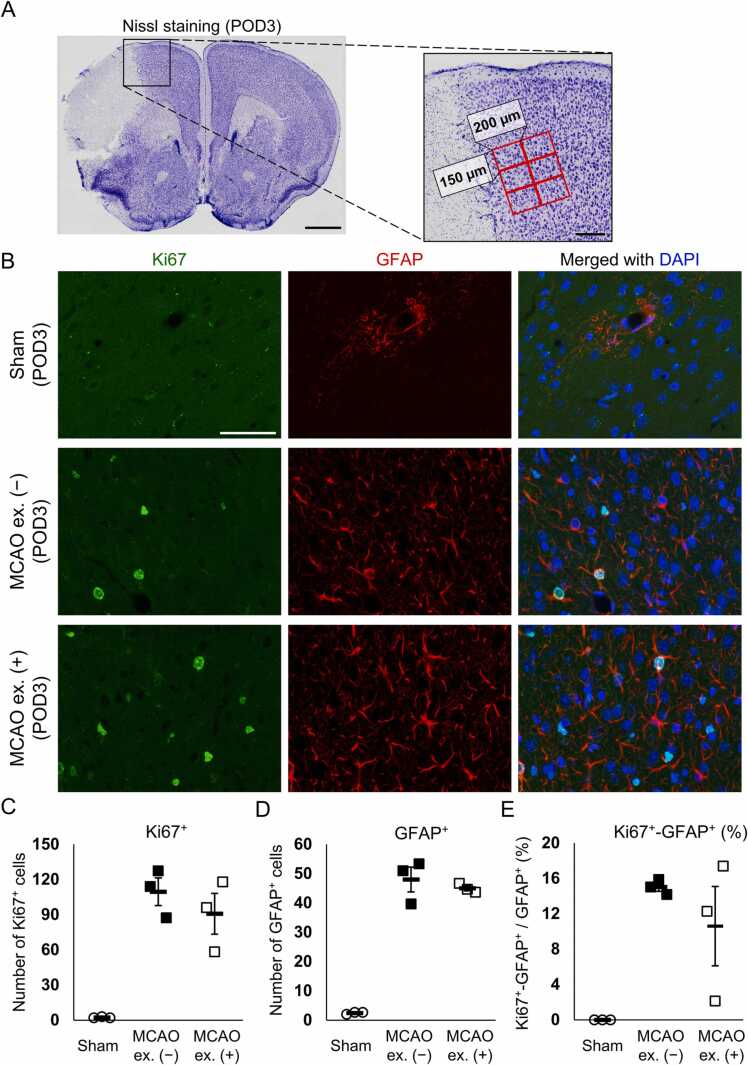
Exercise did not promote proliferation of GFAP-positive cells on POD3. (A) Representative Nissl staining in a POD3 mouse brain. A 450 µm × 400 µm area next to the infarct was analyzed by capturing six positions per section in a field of view of 150 µm × 200 µm. Scale bar: 1 mm (low-magnification image) and 200 µm (high-magnification image). (B) Sections were stained for Ki67 and GFAP on POD3. The infarct boundary is located at the left side of the image. Scale bar: 50 µm. (C-E) Number of Ki67-positive cells (C), GFAP-positive cells (D), and ratio of Ki67 and GFAP double-positive cells to total GFAP-positive cells (E) in the sham, MCAO + non-exercise (MCAO ex. [−]), and MCAO + exercise (MCAO ex. [+]) groups. n = 3 mice in the sham, MCAO ex. (−), and MCAO ex. (+) groups. Dots and squares indicate the average of three sections of individual mice. Bars represent the mean ± standard error of the mean.

Considering that voluntary running exercise increased the ratio of GFAP-positive cells born between POD0 and 3 to the total number of GFAP-positive cells on POD15 (Fig. 1N), we investigated whether exercise enhanced astrocyte proliferation during the acute phase. Post-ischemic proliferation of GFAP-positive cells is predominantly induced around POD3 (Li et al., 2014). Therefore, we counted the cell-cycle related protein Ki67- and GFAP-positive cells in a 450 µm × 400 µm area adjacent to the infarct site on POD3 (Fig. 2A). The cortex of the mice in the sham group showed few Ki67-positive cells, GFAP-positive cells, or Ki67 and GFAP double-positive cells (Fig. 2B–E). In contrast, MCAO increased the number of Ki67-positive cells, GFAP-positive cells, and the ratio of Ki67 and GFAP double-positive cells to the total number of GFAP-positive cells (Fig. 2B–E). However, the ratio of Ki67 and GFAP double-positive cells to the total number of GFAP-positive cells in the MCAO + exercise group did not differ from that in the MCAO + non-exercise group (Fig. 2B and E). Thus, the exercise-induced increase in the population of GFAP-positive astrocytes born between POD0 and 3 on POD15 was probably not owing to the enhanced proliferation in the acute phase after MCAO.

### Exercise did not affect astrocytic cell death after cerebral ischemia

**Fig. 3:**
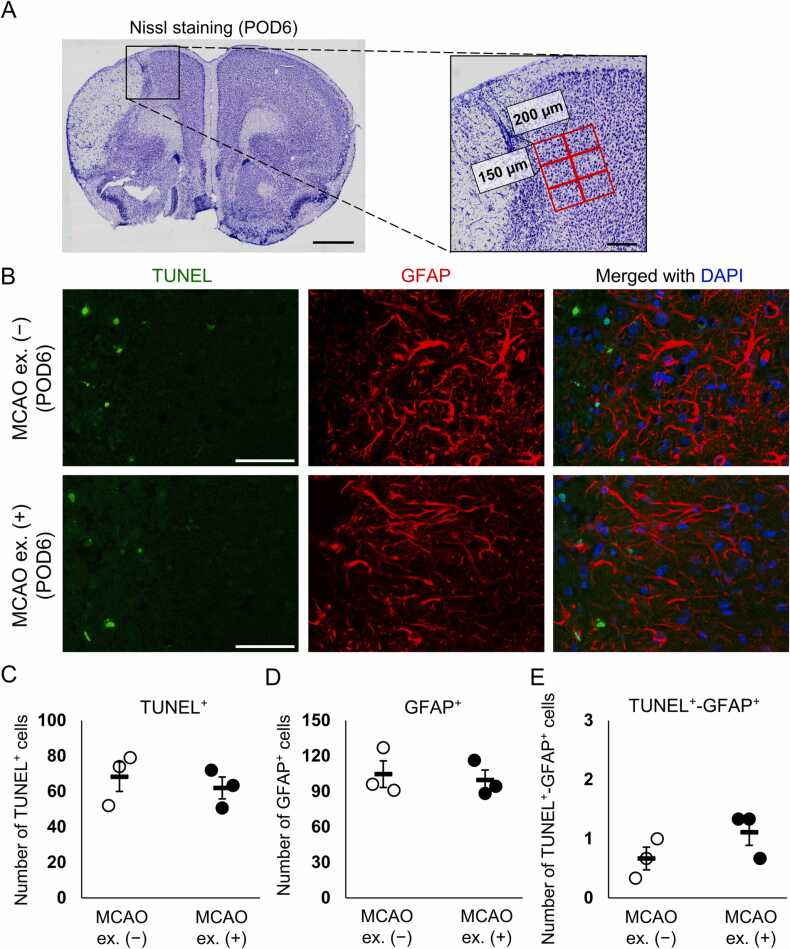
GFAP and TUNEL double-positive cells in the MCAO + non-exercise and MCAO + exercise groups on POD6. (A) Representative Nissl staining in a POD3 mouse brain. A 450 µm × 400 µm area next to the infarct was analyzed by capturing six positions per section in a field of view of 150 µm × 200 µm. Scale bar: 1 mm (low-magnification image) and 200 µm (high-magnification image). (B) Sections were stained for TUNEL and GFAP on POD6. The infarct boundary is located at the left side of the image. Scale bar: 50 µm. (C-D) Number of TUNEL-positive cells (C), GFAP-positive cells (D), and TUNEL and GFAP double-positive cells (E) in the MCAO + non-exercise (MCAO ex. [−]) and MCAO + exercise (MCAO ex. [+]) groups. n = 3 mice in both the MCAO ex. (−) and MCAO ex. (+) groups. Dots and bars represent the data of individual mice and mean ± standard error of the mean, respectively.

Exercise increased the population of GFAP-positive cells born between POD0 and 3 on POD15. However, exercise did not increase the total number of GFAP-positive astrocytes on POD15 or the astrocyte proliferation in the acute phase after MCAO. If exercise inhibited cell death in GFAP-positive astrocytes born between POD0 and 3 and instead increased cell death in residential GFAP-positive astrocytes, this could explain the increased population of GFAP-positive cells born between POD0 and 3 on POD15 without increasing the total number of GFAP-positive astrocytes. A previous study revealed a decrease in the number of GFAP-positive cells after POD6 (Li et al., 2014). Therefore, we performed a TUNEL assay in a 450 µm × 400 µm area adjacent to the infarct site on POD6 (Fig. 3A). TUNEL-positive cells were mainly observed near the infarct border (Fig. 3B). We did not detect differences in the number of GFAP-positive cells, TUNEL-positive cells, or TUNEL and GFAP-double positive cells between the MCAO + non-exercise and MCAO + exercise groups (Fig. 3C–E). Furthermore, the small number of TUNEL and GFAP double-positive cells in both groups (< 1.4 cells in a 450 µm × 400 µm area) suggests that exercise-induced significant increase in the number of cell death in residential GFAP-positive astrocytes and decrease in the number of cell death in GFAP-positive astrocytes born between POD0 and 3 cannot be expected. Thus, the increased population of GFAP-positive cells born between POD0 and 3 on POD15 without increasing the total number of GFAP-positive astrocytes was not owing to the changes in cell death of GFAP-positive cells due to exercise.

Despite the low number of dying GFAP-positive cells (Fig. 3E), the number of GFAP-positive cells in the MCAO + non-exercise and MCAO + exercise groups on POD15 was smaller than those on POD3 (Fig. 1M and 2D). Our results suggest that GFAP-positive cells reduced GFAP expression after POD3. The exercise-induced increase in the population of GFAP-positive astrocytes born between POD0 and 3 without increasing the total number of GFAP-positive astrocytes may be explained by promoting the decrease of GFAP expression in residential astrocytes before ischemia and inhibiting the decrease of GFAP expression in astrocytes born between POD0 and 3.

### Exercise reduced Lcn2 protein expression in astrocytes after cerebral ischemia

**Fig. 4:**
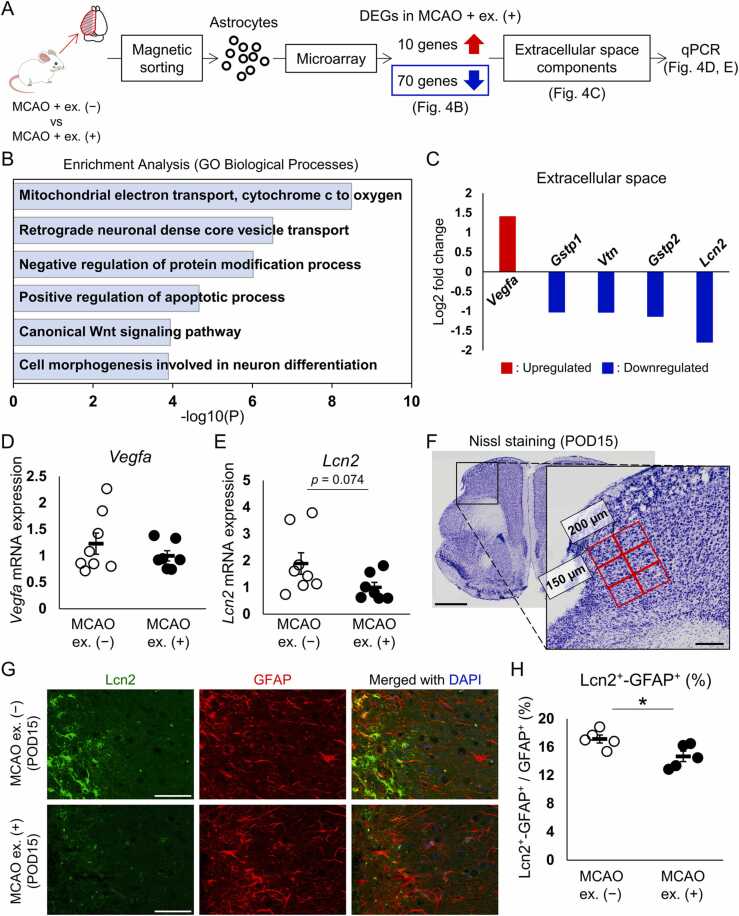
Exercise reduced Lcn2 expression in post-ischemic astrocytes. (A) Astrocytes were sorted from the ipsilateral cortices of the MCAO + non-exercise (MCAO ex. [−]) and MCAO + exercise (MCAO ex. [+]) groups and underwent transcriptome and qPCR analyses. (B) Top six clusters of GO biological process enrichment analysis. (C) DEGs of astrocytes in the MCAO ex. (+) group associated with the GO cellular component term, “extracellular space.” (D, E) Relative expression levels of (D) *Vegfa* and (E) *Lcn2* mRNA in ischemic astrocytes. Target gene expression was normalized to *36b4* expression. The expression levels of the MCAO ex. (−) group were set to 1. Dots and bars represent the individual sample (2 or 3 mice/sample) and mean ± standard error of the mean, respectively. (F) Representative Nissl staining in a POD15 mouse brain. A 450 µm × 400 µm area next to the infarct was analyzed by capturing six positions per section in a field of view of 150 µm × 200 µm. Scale bar: 1 mm (low-magnification image) and 200 µm (high-magnification image). (G) Sections were stained for Lcn2 and GFAP on POD15. The infarct boundary is located at the left side of the image. Scale bar: 50 µm. (H) Ratio of Lcn2 and GFAP double-positive cells to total GFAP-positive cells in the MCAO + non-exercise (MCAO ex. [−]) and MCAO + exercise (MCAO ex. [+]) groups. n = 5 mice in both the MCAO ex. (−) and MCAO ex. (+) groups. Dots and bars represent the data of individual mice and mean ± standard error of the mean, respectively. * *p* < 0.05.

Exercise increased the population of GFAP-positive astrocytes born between POD0 and 3 on POD15 (Fig. 1N) while ameliorating ischemia-induced dendritic spine loss on POD15 (Yamaguchi et al., 2021). Synaptic remodeling is influenced by the perineuronal environment (Eroglu and Barres, 2010, Song and Dityatev, 2018), and astrocytes contribute to its formation. Therefore, we expected that the population change in GFAP-positive astrocytes by exercise would be accompanied by functional changes on POD15. To investigate the molecular changes in astrocytes, the astrocytic transcriptome of the MCAO + exercise group was compared with that of cells in the MCAO + non-exercise group on POD15. Astrocytes were sorted from the ipsilateral cortices of the mice in the MCAO + non-exercise and MCAO + exercise groups. Ten upregulated (Table 1) and 70 downregulated genes (Table 2) were detected in the MCAO + exercise group compared to the MCAO + non-exercise group (accession number GSE210674, https://www.ncbi.nlm.nih.gov/geo/query/acc.cgi?acc=GSE210674) (Fig. 4 A). The GO biological processes term “Cell morphogenesis involved in neuron differentiation” associated with the downregulated genes in astrocytes in the MCAO + exercise group was included in the top-ranked categories (Fig. 4B). Exercise inhibits MCAO-induced dendritic spine loss (Yamaguchi et al., 2021). To identify the astrocytic factors interacting with neurons, secreted type factors annotated to the GO cellular component term “extracellular components” were isolated from the DEGs. Consequently, one upregulated gene (*Vegfa*) and four downregulated genes [*Lcn2*; *Glutathione S-transferase, pi 2* (*Gstp2*); *Vitronectin* (*Vtn*); and *Glutathione S-transferase, pi 1* (*Gstp1*)] were identified (Fig. 4C). The most upregulated (*Vegfa*) and most downregulated (*Lcn2*) genes are involved in dendritic spine density modulation; VEGFA induces dendritic spine formation (Licht et al., 2010) whereas Lcn2 reduces dendritic spine density (Mucha et al., 2011). The expression of *Vegfa* and *Lcn2* in post-ischemic astrocytes was examined by qPCR because microarray data normalization can induce overestimation (Wang et al., 2011). We confirmed a tendency for decreased *Lcn2* expression after exercise (*p* = 0.074) (Fig. 4D and E). Furthermore, Lcn2 protein expression was analyzed in a 450 µm × 400 µm area adjacent to the infarct on POD15 (Fig. 4F). Lcn2 expression was mainly observed in the area near the infarct border and some Lcn2 signals were merged with GFAP signals (Fig. 4G). Moreover, there were more GFAP and Lcn2 double-positive cells in the deep cortex layers (Fig. 4G). We confirmed that exercise significantly decreased the ratio of Lcn2 and GFAP double-positive cells to the total number of GFAP-positive cells (*p* = 0.031), and Lcn2 staining intensity in GFAP-positive cells in the MCAO + exercise group was weaker than that in the MCAO + non-exercise group (Fig. 4G and H). Thus, exercise substantially downregulated the protein level of Lcn2 production in GFAP-positive cells. These results suggest that exercise modifies astrocytic phenotype after cerebral ischemia.

**Table 1:** The upregulated genes in MCAO + exercise astrocytes.

Gene symbol	Log2 fold change
Gm2310	1.461957553
Gm10974	1.451961352
Vegfa	1.401180263
Gm17428	1.398029393
Snrnp70	1.336465849
Alcam	1.098251221
Gm11060	1.092815175
Gm21738	1.067646042
H2-Ea-ps	1.059989931
Ms4a7	1.037801654

**Table 2:** The downregulated genes in MCAO + exercise astrocytes.

Gene symbol	Log2 fold change
Kif5a	-4.087966618
Camk2a	-3.127336747
Trp53inp2	-2.880754141
Cdr1	-2.653389013
Rcan2	-2.432248794
Sepw1	-2.421518197
Bcas1	-2.419597862
Kif1a	-2.333921402
Kif5b	-2.225187027
Gpm6a	-2.119140636
Mobp	-2.063638014
Pkp4	-2.014174867
Scd1	-1.971750236
Ptpro	-1.914882812
Dync1li2	-1.805539875
Snrpn	-1.791156729
Lcn2	-1.789195533
Zfp365	-1.788642587
Cox6a1	-1.747299392
Lpar1	-1.694715194
Cox6c	-1.667423475
Ndufb9	-1.656065148
Plekhb1	-1.614326348
Ankrd12	-1.524885052
Tmem47	-1.482622292
Map4	-1.469505217
Cox8a	-1.434944875
Apc	-1.404991548
Rps21	-1.402470826
Cyb5r3	-1.389229017
Rhou	-1.374908091
Qdpr	-1.370572773
Hipk2	-1.36697422
Fam213a	-1.35645669
Usmg5	-1.346652579
Sirt2	-1.336671615
Rpl37rt	-1.318596157
Cox4i1	-1.314964189
Nudt4	-1.282897841
Slc1a3	-1.257186671
Stmn1	-1.249932909
Aldoc	-1.23955907
Fads2	-1.233734297
Calm3	-1.219045282
Fam171b	-1.166730727
Odc1	-1.165191612
Klf4	-1.161459177
Dusp3	-1.155628649
Stau2	-1.154947283
Plpp3	-1.136989764
Gstp2	-1.132053582
Erbb2ip	-1.115016961
Ednrb	-1.112510909
Rab6a	-1.098184885
Cryab	-1.078615066
Ddit3	-1.06438597
Pacs2	-1.06110574
Aes	-1.060375329
Nudt3	-1.05254725
Rps7	-1.034765276
Vtn	-1.025434486
Cox5b	-1.023273515
Gstp1	-1.021524444
Ndrg1	-1.019337145
Pea15a	-1.016534917
Tppp	-1.015346073
1700047I17Rik2	-1.011476647
Rps2	-1.01120742
Sccpdh	-1.011068192
Myeov2	-1.002075601

## Discussion

We previously demonstrated that the number of dendritic spines in the peri-infarct motor cortex of the MCAO + exercise mice was larger than that of the MCAO + non-exercise mice (Yamaguchi et al., 2021). Moreover, the perineuronal environment influences synaptic function and structure. Therefore, we focused on the exercise-induced changes in non-neuronal cells following cerebral ischemia.

**Fig. 5:**
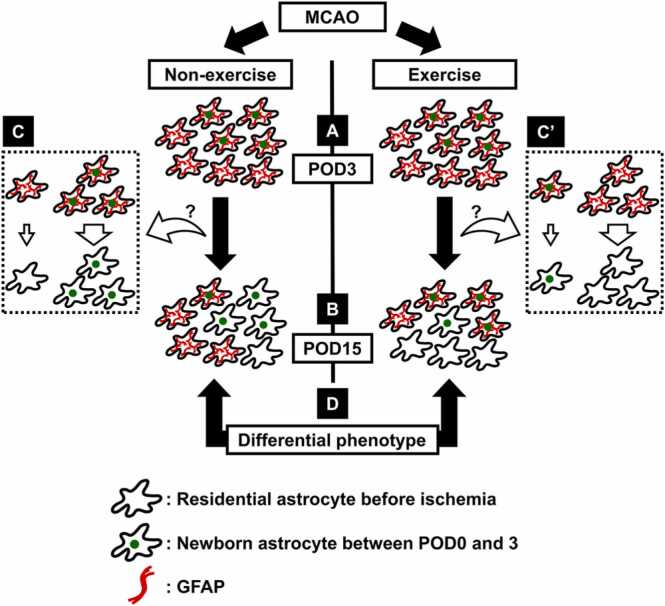
Schematic representation of the effect of exercise on post-ischemic astrocytes. (A) Exercise does not increase the population of newborn GFAP-positive astrocytes on POD3. (B) On the other hand, exercise increases the population of GFAP-positive astrocytes born between POD0 and 3 on POD15. However, exercise does not increase the total number of GFAP-positive astrocytes on POD15. Furthermore, despite the low number of dying GFAP-positive astrocytes, the number of GFAP-positive astrocytes is decreased after POD3. (C, C’) Hence, we believe that GFAP-positive astrocytes reduce their GFAP expression. Exercise-induced increase in the population of GFAP-positive astrocytes born between POD0 and 3 without increasing the total number of GFAP-positive astrocytes may be explained by promoting the decrease of GFAP expression in residential astrocytes before ischemia and inhibiting the decrease of GFAP expression in astrocytes born between POD0 and 3. (D) The astrocytic cluster affected by exercise exhibits a differential phenotype from that derived from lack of exercise.

Glial cells upregulate their proliferative activity in the ischemic cortex (Li et al., 2014, Sawano et al., 2019, Sawano et al., 2015, Wada et al., 2016), which affects the perineuronal environment. Post-ischemic glial proliferation in the peri-infarct area has been reported to peak around POD3 (Li et al., 2014, Sawano et al., 2015). Therefore, we investigated the effect of exercise on the late-phase existence of glial cells born between POD0 and 3 using BrdU labeling. Exercise increased the population of GFAP-positive astrocytes born between POD0 and 3, without increasing the total number of GFAP-positive astrocytes on POD15 (Fig. 1M and N). Ki67 staining indicated that exercise did not promote proliferation of GFAP-positive astrocytes on POD3 (Fig. 2E). Thus, the exercise-related increase in the population of GFAP-positive astrocytes born between POD0 and 3 on POD15 was neither due to a corresponding increase in the number of post-mitotic GFAP-positive astrocytes nor due to changes in cell death of GFAP-positive astrocytes, as few GFAP-positive cells died after cerebral ischemia, and no differences in the number of TUNEL and GFAP double-positive cells between the MCAO + non-exercise and MCAO + exercise groups were observed (Fig. 3E). Thus, exercise did not affect the GFAP-positive astrocytic population through cell death; however, it is possible that exercise changed GFAP expression in post-ischemic astrocytes. The number of GFAP-positive cells increased in the acute phase of cerebral ischemia and decreased after POD6 (Fig. 2B, E) (Li et al., 2014). However, death of GFAP-positive cells was hardly observed on POD6 (Fig. 3E). A fate tracing experiment using *GFAP-CreER*^*TM*^*;tdRFP* mice administrated tamoxifen after MCAO demonstrated that approximately 25% of tdRFP-positive cells lack immunoreactivity for GFAP (Shimada et al., 2012), indicating that GFAP-positive astrocytes reduce their GFAP expression after ischemia. Based on these results, we believe that exercise inhibited the decrease in GFAP expression in BrdU-incorporated astrocytes while promoted the decrease in GFAP expression in residential astrocytes before ischemia, resulting in an increase in the population of BrdU and GFAP double-positive astrocytes without increasing the total number of GFAP-positive astrocytes (Fig. 5). A previous study indicated a decreased number of GFAP-positive cells after POD6 (Li et al., 2014), implying that exercise changes GFAP expression approximately 1 week after MCAO. The running distances of four of five mice reached > 1 km within 24 h after MCAO and gradually increased after POD8 (Fig. 1B). Thus, we consider that the change in GFAP expression was induced by the synergistic effect of continuous running exercise for ≥ 1 week and high-intensity exercise approximately 1 week after MCAO.

Our results imply that exercise alters GFAP expression after cerebral ischemia. GFAP is a marker of reactive astrocytes (Eng and Ghirnikar, 1994), and this change in GFAP expression implies a change in astrocytic activation. Some subtypes of reactive astrocytes have different transcriptomes and functions (Liddelow et al., 2017, Zamanian et al., 2012), and their phenotypes vary depending on the situation (Zamanian et al., 2012). Exercise subtly decreased *Lcn2* expression in post-ischemic astrocytes (Fig. 4E). Previous studies demonstrated that the translation of Lcn2 is directly inhibited by miR-138 (Xiong et al., 2016), which is upregulated in the injured cerebral cortex by exercise (Miao et al., 2015). Consistently, immunohistological analysis revealed that exercise decreased the number of Lcn2-positive astrocytes on POD15 (Fig. 4H). Lcn2, a secretory protein belonging to the lipocalin family, is upregulated in astrocytes and endothelial cells by ischemia and inflammatory stimuli (Jin et al., 2014, Suk, 2016, Wan et al., 2022), whereas its receptor is mainly expressed in neurons, astrocytes, and endothelial cells (Jin et al., 2014). The addition of Lcn2 to primary neurons reduces dendritic spine density (Mucha et al., 2011), and *Lcn2*-deficient mice show higher dendritic spine density (Skrzypiec et al., 2013). Furthermore, Lcn2 induces pro-inflammatory activation in astrocytes (Jang et al., 2013, Lee et al., 2009). Inflammatory stimuli cause dendritic spine loss and synaptic dysfunction (Centonze et al., 2009, Prada et al., 2018, Tong et al., 2012). Therefore, Lcn2 contributes to the reduction of dendritic spines. Previous research showed that Lcn2 induction peaks at 24 h after MCAO (Jin et al., 2014). However, we found Lcn2-positive astrocytes in the region near the infarct border, especially in the deep cortical layers on POD15 (Fig. 4G). Consistently, we observed dendritic spine loss inhibition in the peri-infarct cortex layer 5 induced by exercise in our previous study (Yamaguchi et al., 2021). Furthermore, Lcn2 promotes demyelination by astrocytic myelin phagocytosis after ischemia, while its inhibition leads to the amelioration of ischemic injury and behavioral abnormalities (Jin et al., 2014, Wan et al., 2022). These data suggest that exercise decreased Lcn2 expression in astrocytes, contributing to the inhibition of dendritic spine loss and recovery of motor function after cerebral ischemia (Yamaguchi et al., 2021).

This study has two major limitations that need to be addressed in future studies. First, we could not determine whether the differences in astrocytes between the MCAO + non-exercise and MCAO + exercise groups were caused by the effect of exercise alone or by the interactive effect of exercise and cerebral ischemia. Second, we could not determine whether the exercise-induced downregulation of Lcn2 expression in post-ischemic astrocytes was dependent on post-ischemic proliferation; however, in any case, exercise affects astrocytic population and phenotype in the peri-infarct cortex. Furthermore, we expected phenotypic differences between newborn astrocytes after ischemia and residential astrocytes before ischemia. Few studies have focused on the differences in the features between pre- and post-mitotic astrocytes. We believe that our data provides a new approach for revealing novel astrocytic functions.

In summary, our results show that voluntary running exercise increases the population of GFAP-positive astrocytes born between POD0 and 3 on POD15 in the peri-infarct cortex. Changes in Lcn2 expression in GFAP-positive astrocytes indicate that exercise alters astrocytic features after cerebral ischemia.

## Funding

This work was supported by JST SPRING (Grant Number JPMJSP2101), 10.13039/100007449Takeda Science Foundation, JSPS KAKENHI (Grant Numbers JP18K15124 to T.S., JP21K15423 to T.S., and JP20K11520 to H.T.).

## CRediT authorship contribution statement

**Natsumi Yamaguchi:** Conceptualization, **i**nvestigation, data curation, formal analysis, writing of the original draft, and funding acquisition. **Toshinori Sawano**: Conceptualization, investigation, data curation, writing of the original draft, funding acquisition, and supervision. **Jin Nakatani:** Investigation, data curation, and writing of the original draft. **Akiko Nakano-Doi:** Methodology. **Takayuki Nakagomi:** Methodology. **Tomohiro Matsuyama:** Methodology. **Hidekazu Tanaka:** Conceptualization, writing of the original draft, funding acquisition, and supervision.

## Conflict of interest

None.
